# Supplementary material for: Spatial transcriptomics reveals alterations in perivascular macrophage lipid metabolism in the onset of Wooden Breast myopathy in broiler chickens
Source: Sci Rep. 2024 Feb 11;14:3450. doi: 10.1038/s41598-024-53904-5 (PMC10859375; doi:10.1038/s41598-024-53904-5)
Supplement: Supplementary file 1 — Supplementary Figures. [file 41598_2024_53904_MOESM1_ESM.docx]

1.
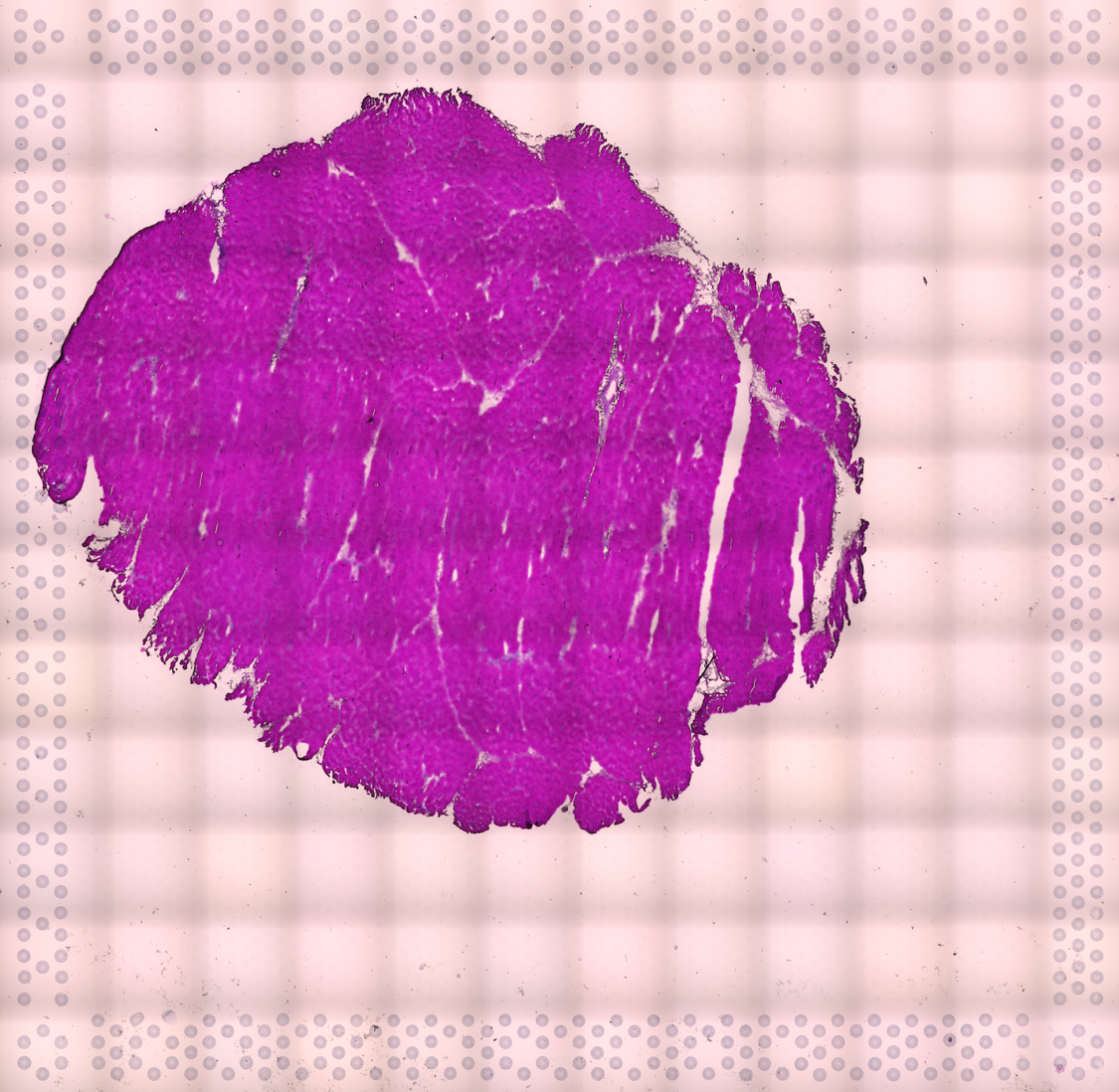

2.
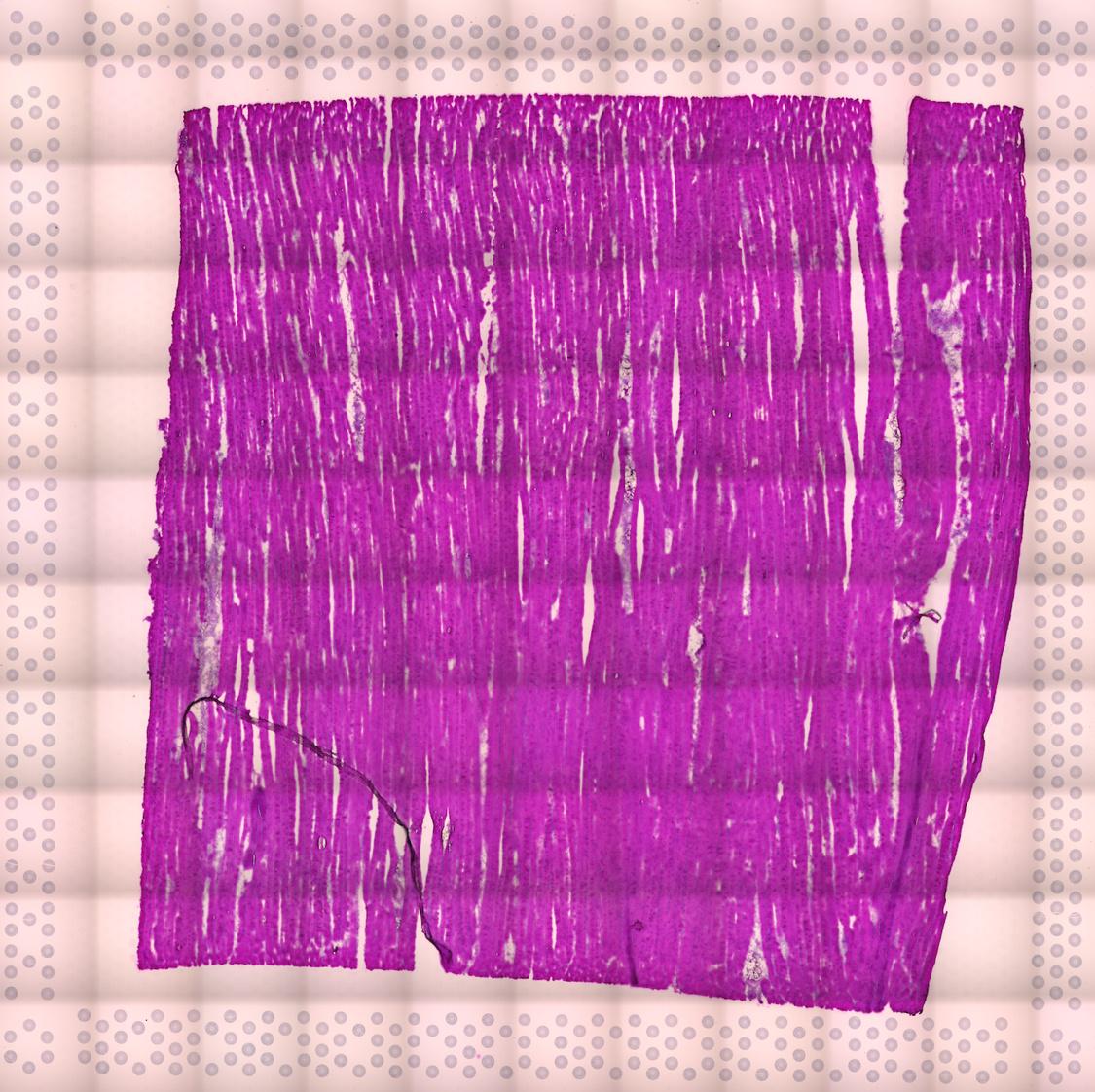

3.
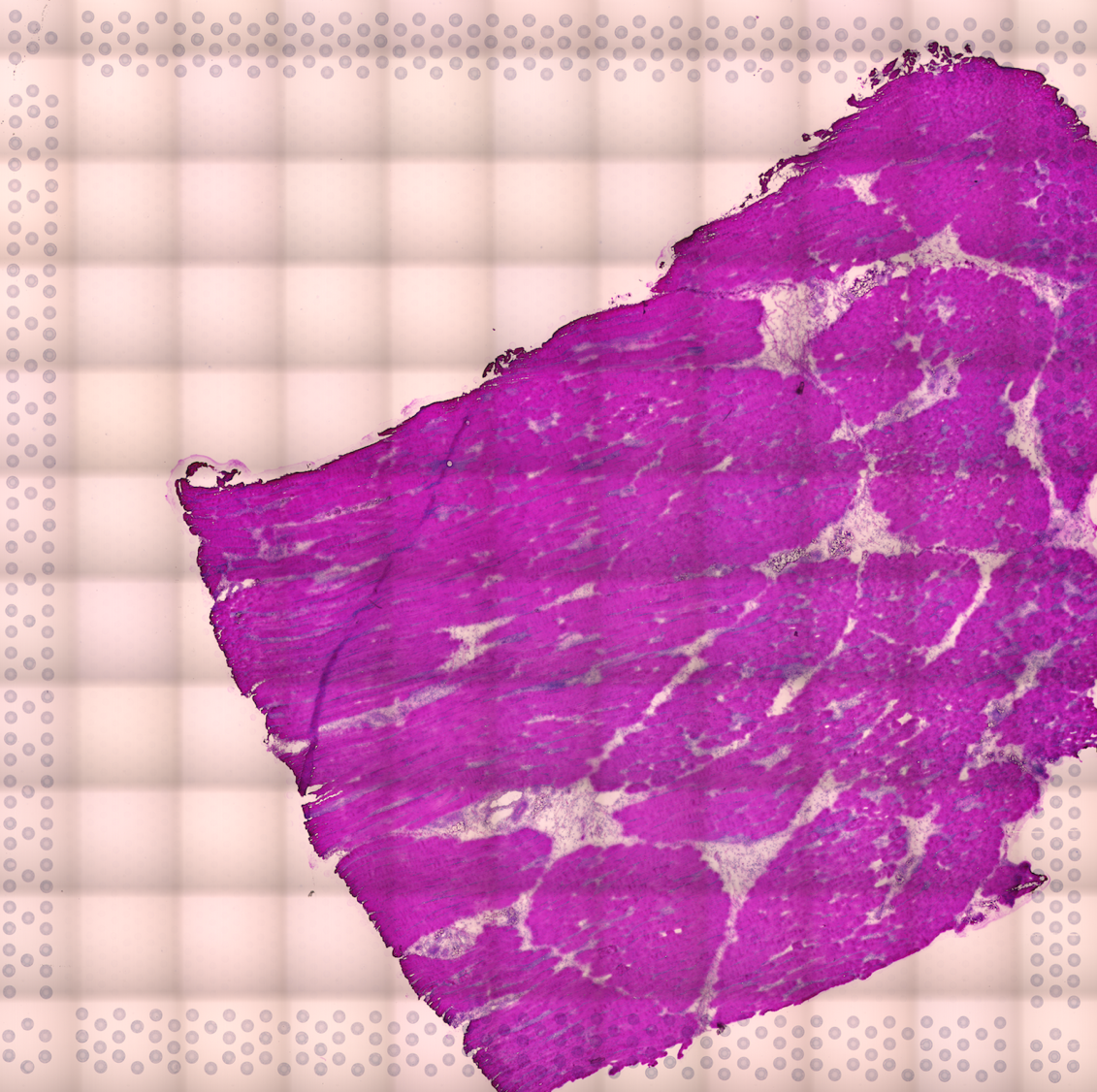

4.
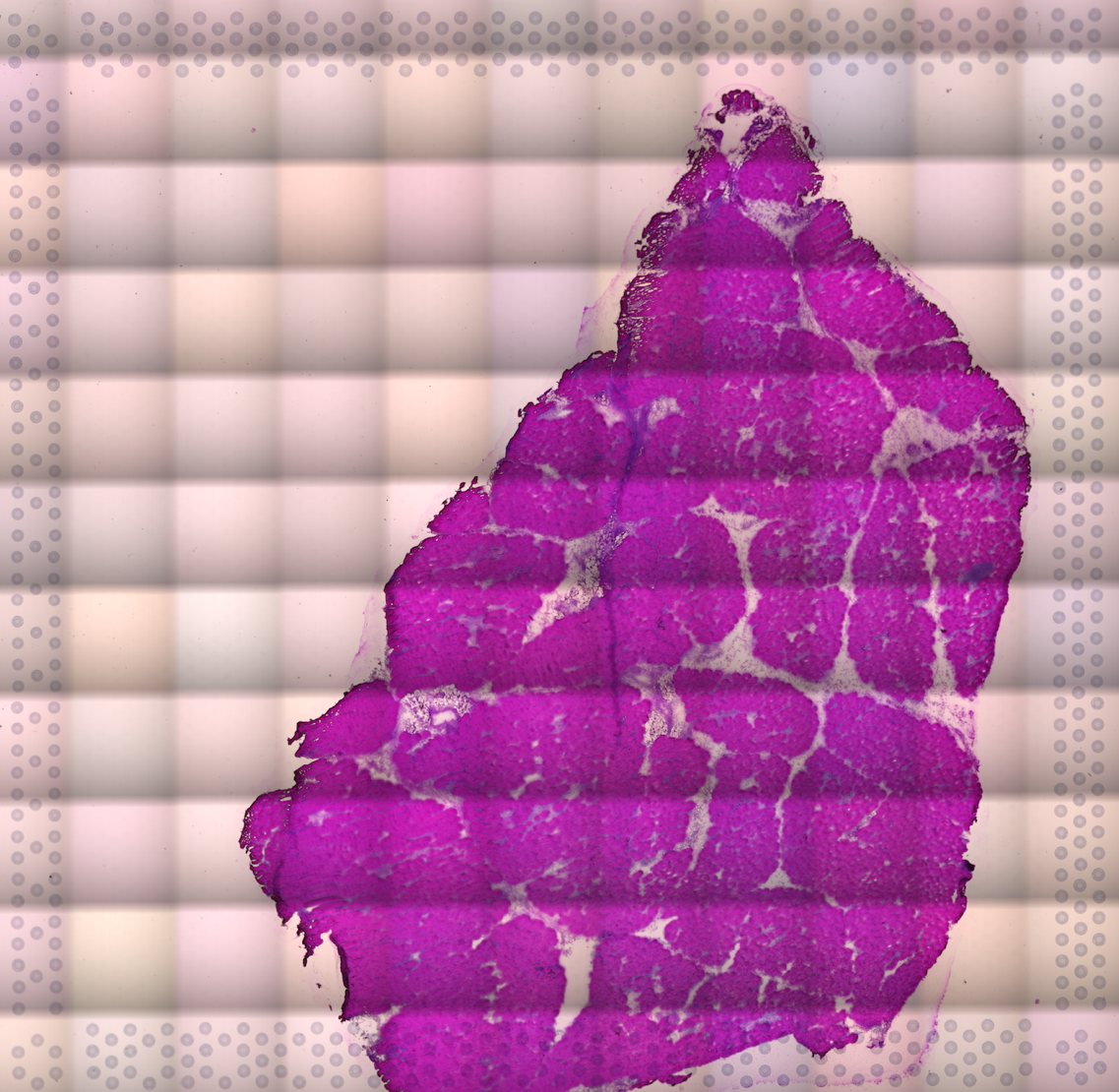


Fig S1. Tile images for samples. A) Sample S1; B) Sample S2; C) Sample S3_1; D) Sample S3_2;


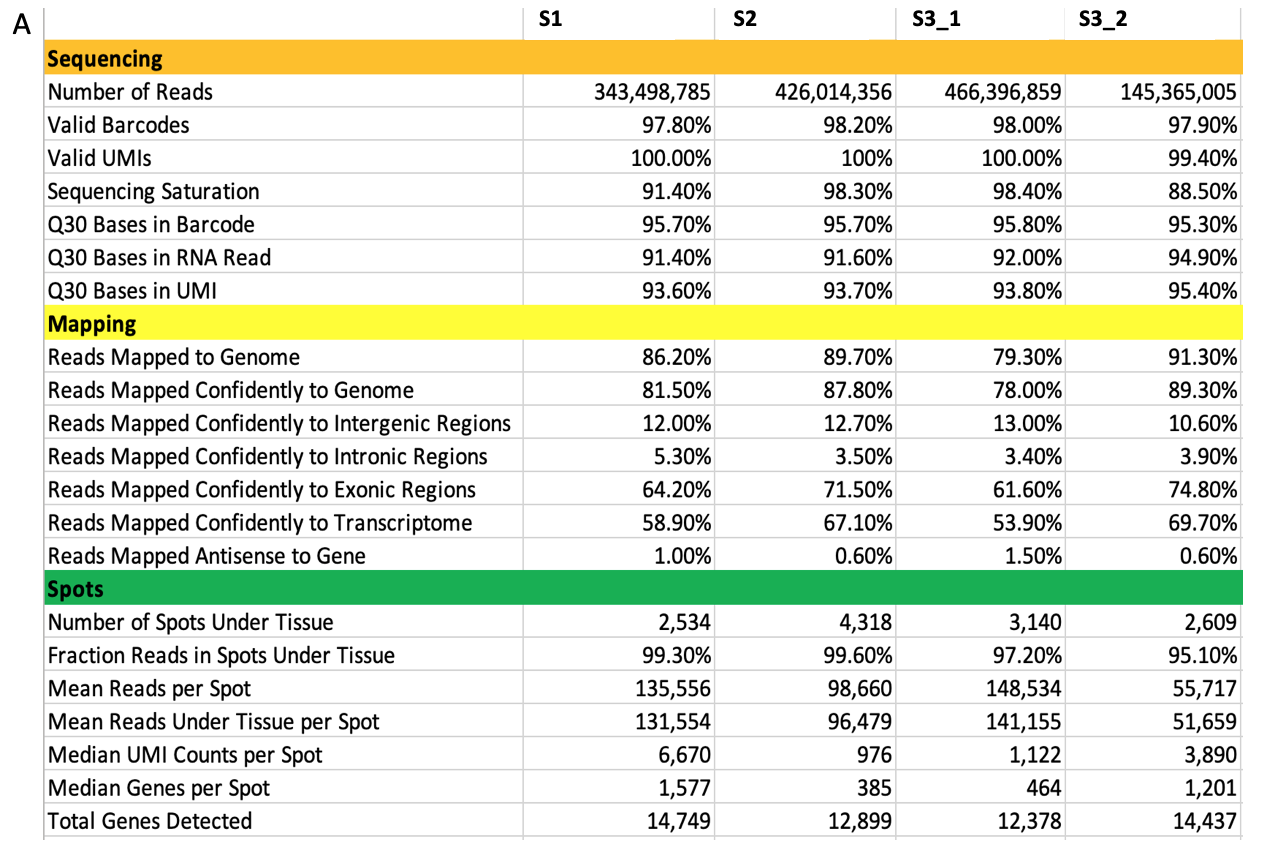


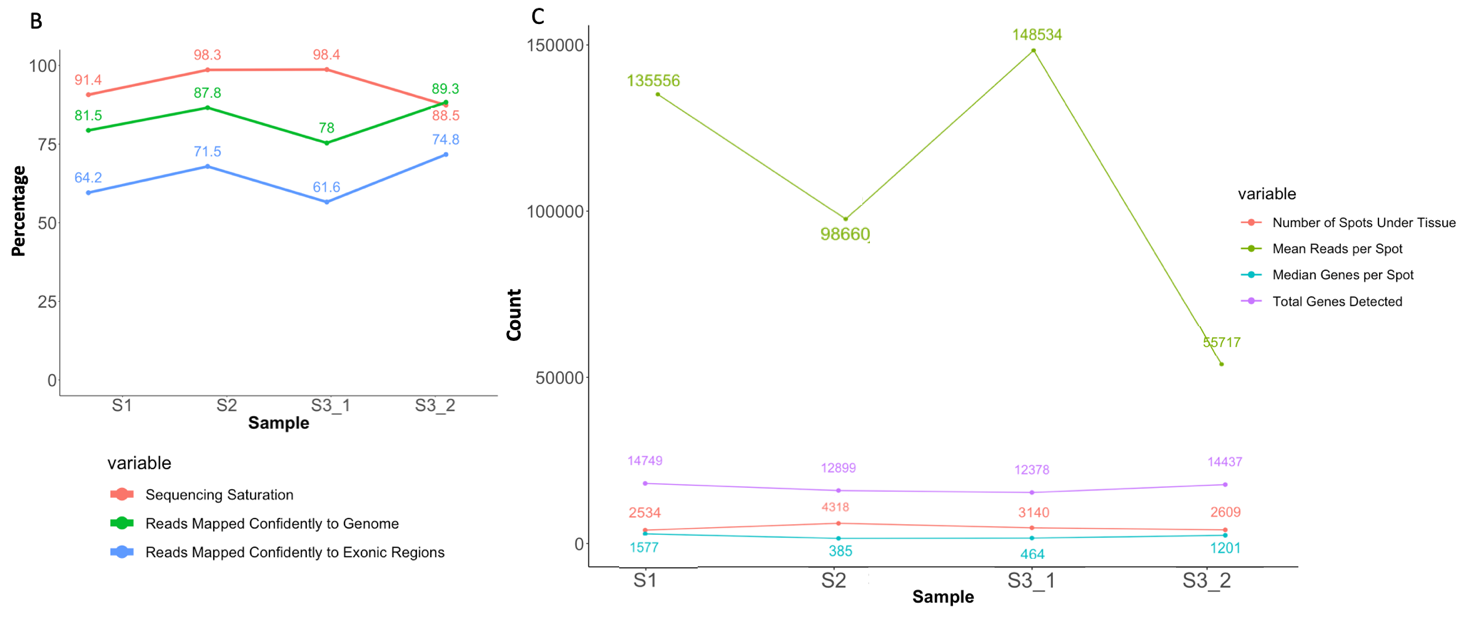


Fig S2. **A)** Sequencing and Mapping results. **B)** Sequencing saturation and mapping percentage of reads from broiler chicken pectoralis major muscle mapped confidently to the chicken genome. **C)** Statistics of number of reads, spots and genes per spot.

S1: sample 1 (oblique); S2: sample 2 (longitudinal); S3_1: sample 3 processed with permeabilization time of 18 minutes (oblique); S3_2: sample 3 processed with permeabilization time of 6 minutes (oblique). S1 and S2 samples were classified as “Unaffected” and Samples S3_1 and S3_2 were classified as “Wooden Breast affected” by routine histologic analysis.


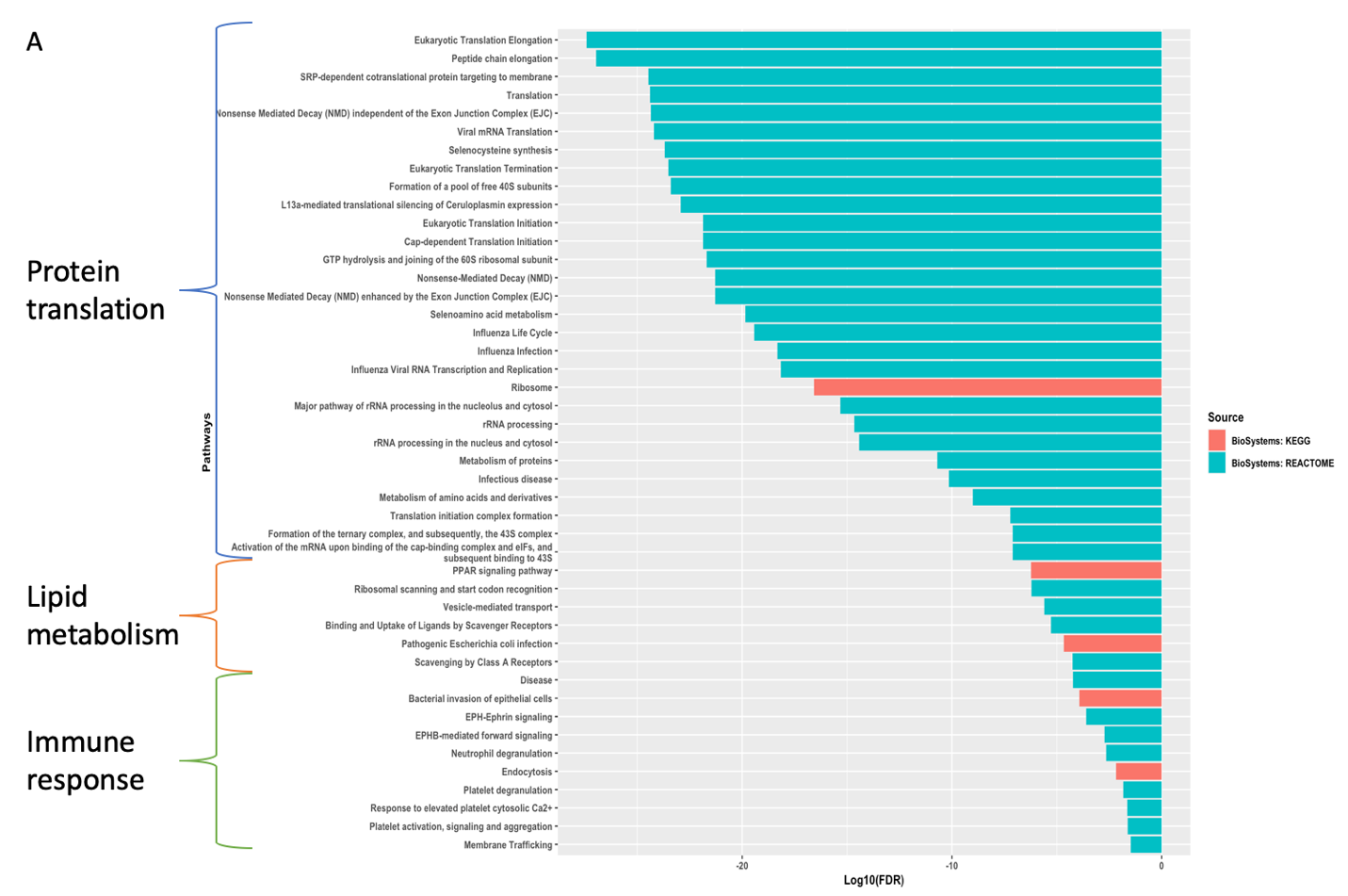


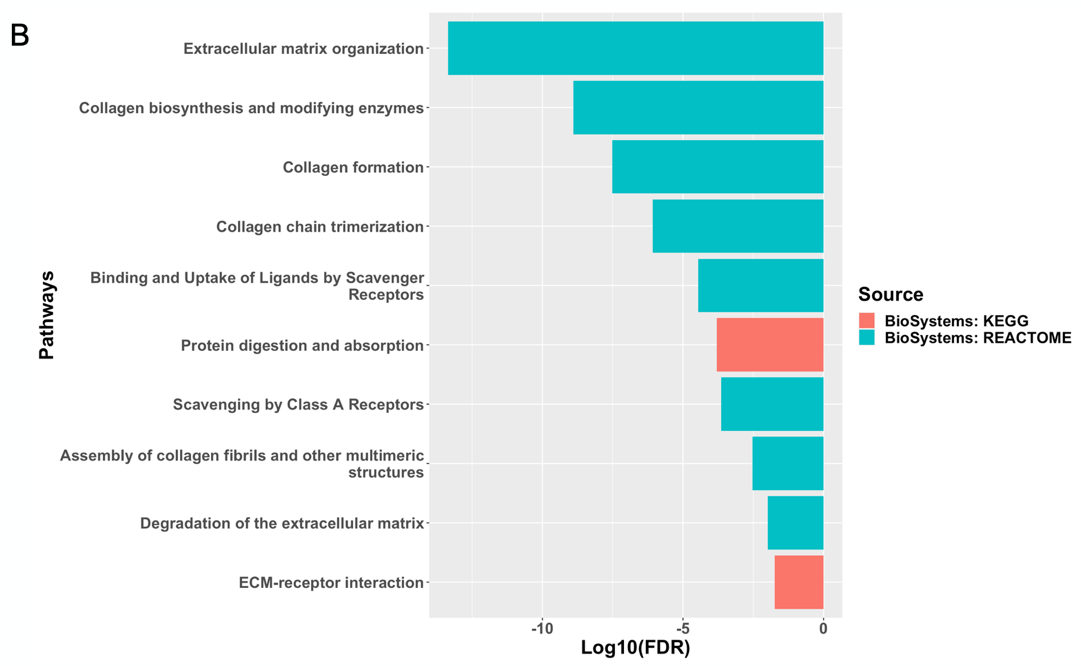


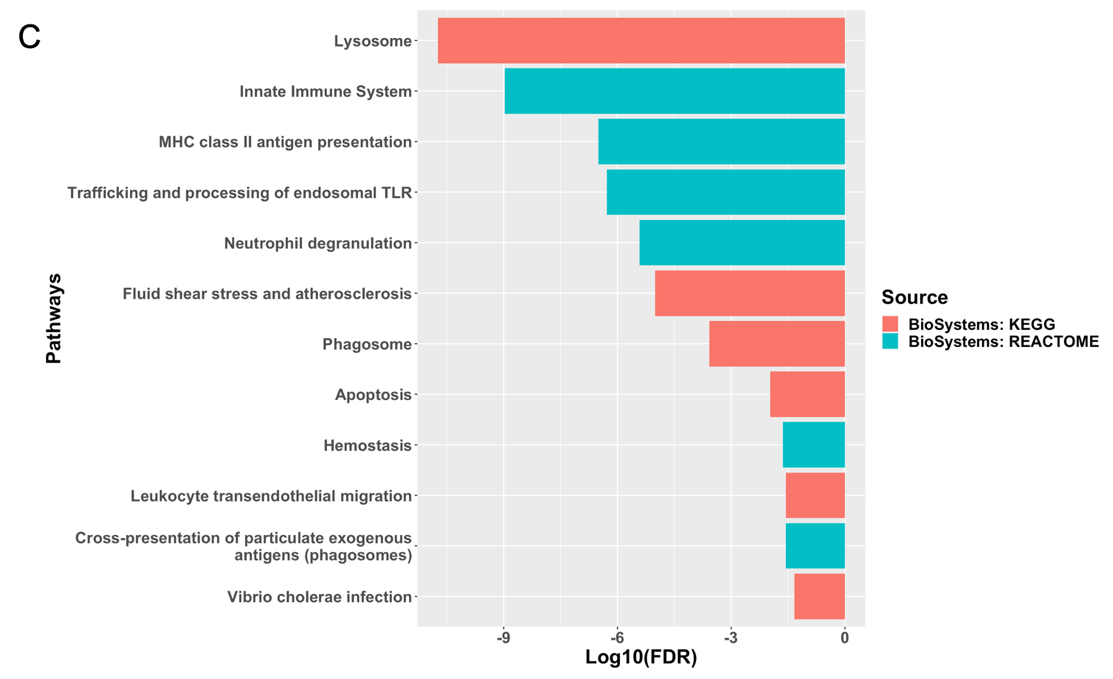


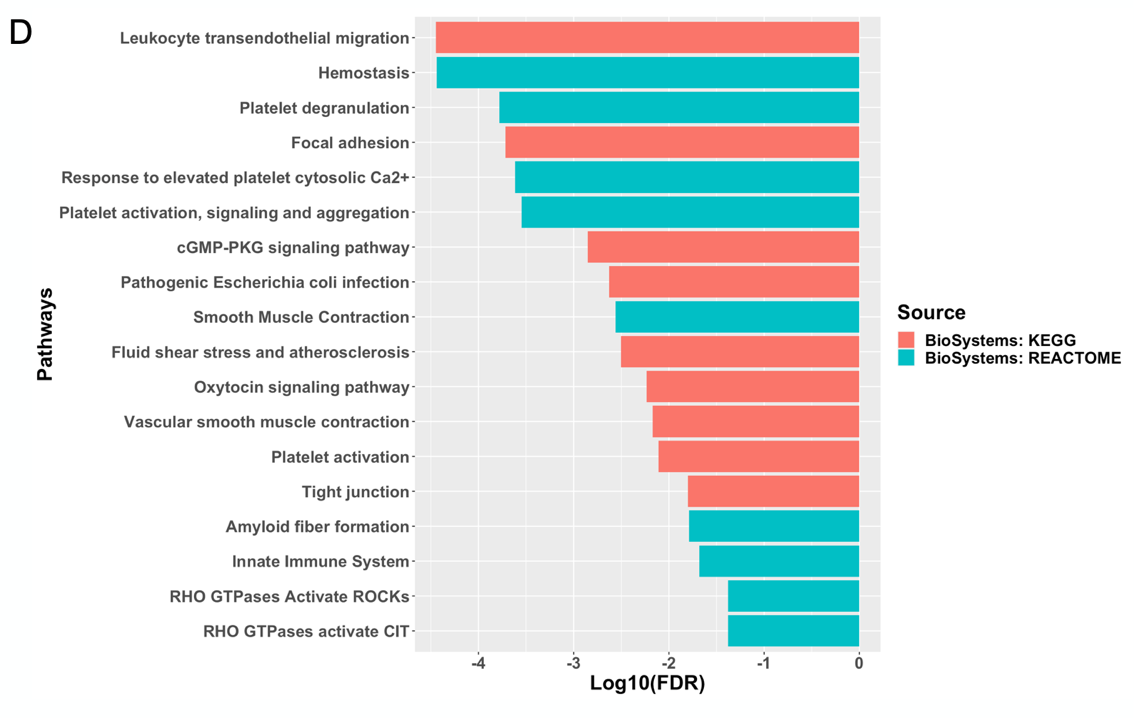


Figure S3: Toppgene enrichment analysis for A: foam cells; B: connective tissue; C: myositis; D: Vascular regions.


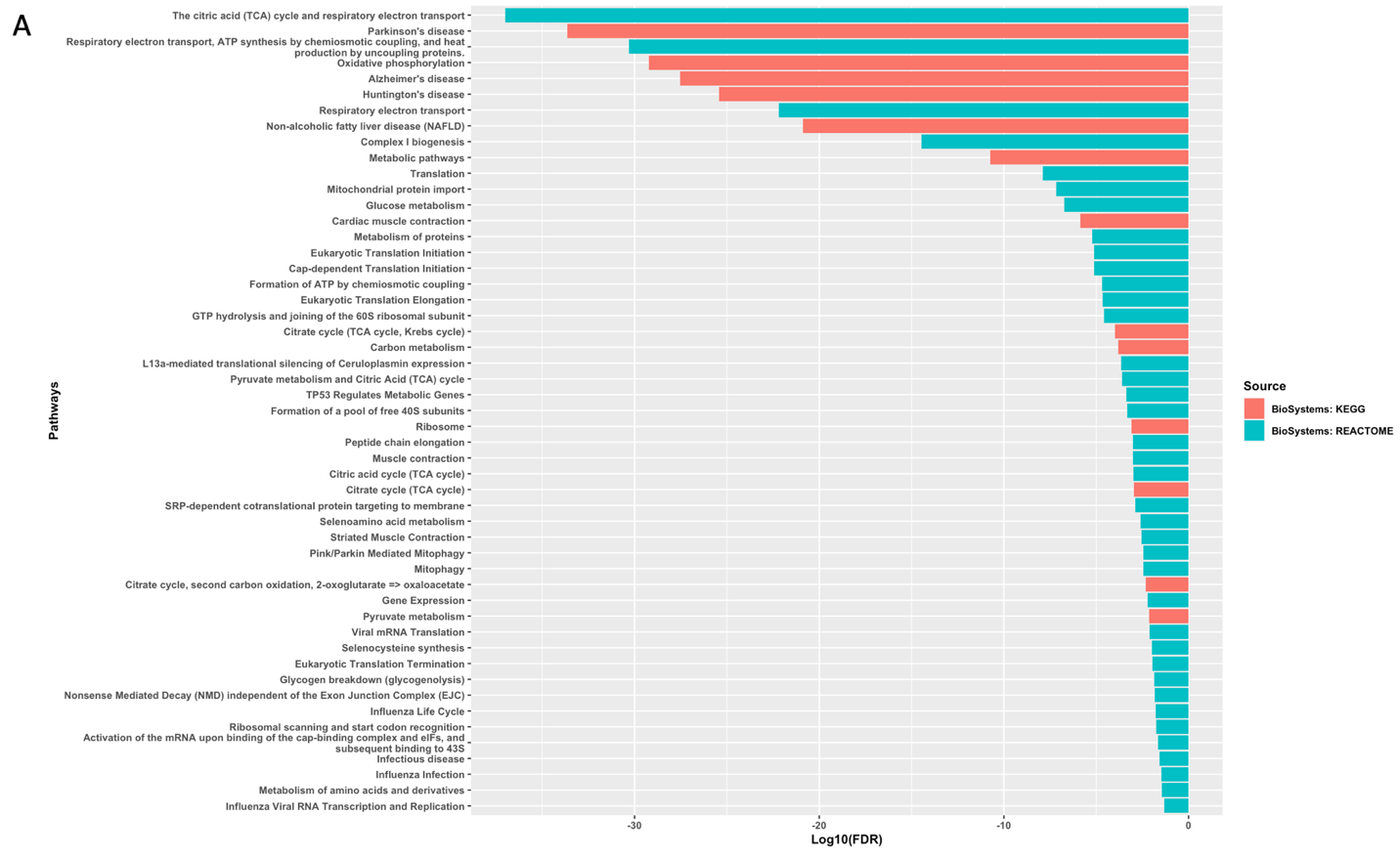


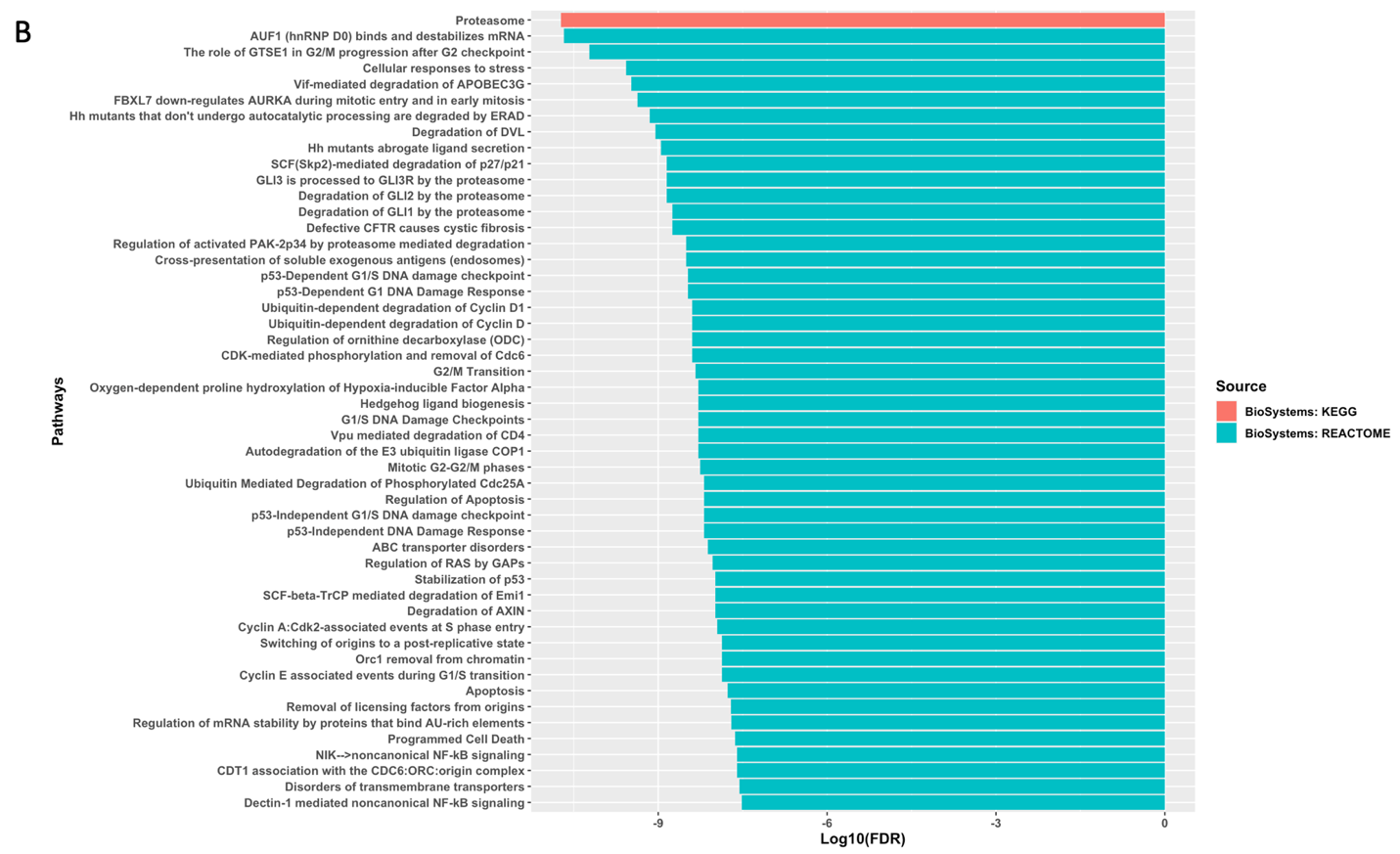


Figure S4: Toppgene enrichment analysis for muscle clusters, A: M2, B: M4 (top 50).
